# Dietary Vitamin B6 Intake and Gastroesophageal Reflux Disease: A Case–Control Study

**DOI:** 10.1002/jgh3.70398

**Published:** 2026-04-14

**Authors:** Amirhesam Mehrara, Saba Moradi, Masoumeh Dorosti, Leila Dehghanian, Masoumeh Sadat MirShafaie, Mohammadreza Esfahanian, Bita Moradi, Ali Nouri, Sara Khoshdooz, Parsa Bahmani, Saeid Doaei, Maryam Gholamalizadeh

**Affiliations:** ^1^ School of Medicine Guilan University of Medical Sciences Rasht Iran; ^2^ Departmant of Medicine Qazvin University of Medical Sciences Qazvin Iran; ^3^ Student Research Committee, Department of Nutrition, Faculty of Medicine Urmia University of Medical Sciences Urmia Iran; ^4^ Shahid Beheshti University of Medical Science, School of Nutrition & Food Technology, National Nutrition & Food Research Institute Tehran Iran; ^5^ Department of Sport Physiology, Tonekabon Branch Islamic Azad University Tonekabon Iran; ^6^ Nursing and Midwifery Department Kermanshah University of Medical Sciences Kermanshah Iran; ^7^ Department of Clinical Nutrition, School of Nutritional Sciences and Dietetics Tehran University of Medical Science Tehran Iran; ^8^ Faculty of Medicine Guilan University of Medical Science Rasht Iran; ^9^ Cancer Research Center Shahid Beheshti University of Medical Sciences Tehran Iran; ^10^ Faculty of Nutrition Sciences and Food Technology, National Nutrition and Food Technology Research Institute Shahid Beheshti University of Medical Sciences Tehran Iran

**Keywords:** B6, dietary intake, gastroesophageal reflux disease, GERD, vitamin B6

## Abstract

**Background:**

Gastroesophageal reflux disease (GERD) is a common gastrointestinal disorder influenced by various dietary factors. This study aimed to evaluate the association between dietary intake of vitamin B6 and the risk of GERD.

**Methods:**

This case–control study included 150 women with GERD and 300 controls in Tehran, Iran. Dietary intake of vitamin B6 was assessed using a validated food frequency questionnaire (FFQ) and analyzed with Nutritionist IV software. Logistic regression models were applied to determine the association between GERD risk and dietary intake of vitamin B6, adjusting for age, education, marital status, occupation, physical activity, BMI, and calorie intake.

**Results:**

Compared with participants in the lowest tertile of vitamin B6 intake, those in the highest tertile had significantly higher odds of GERD in age‐adjusted models (OR = 1.98, 95% CI: 1.12–3.51) and this association remained significant after further adjustment for sociodemographic factors, physical activity, BMI (OR = 2.05, 95% CI: 1.10–3.83), and total energy intake (OR = 2.02, 95% CI: 1.06–3.81). No significant association was observed for the middle tertile.

**Conclusion:**

The findings of this case–control study indicate a positive association between higher dietary vitamin B6 intake and the risk of GERD. Further prospective research would be helpful to confirm these observations and to shed light on possible biological mechanisms.

## 1 Introduction

Gastroesophageal reflux disease (GERD) is a prevalent gastrointestinal disorder characterized by the chronic retrograde movement of gastric contents from the stomach into the esophagus, oral cavity, or lungs. This condition may lead to several complications, including esophageal stricture and esophageal adenocarcinoma [1, 2]. Current estimates indicate that GERD is a highly prevalent chronic gastrointestinal disorder worldwide. A systematic review by Nirwan et al. showed that the global prevalence of GERD is 13.98%, with the highest prevalence reported in Turkey (22.4%) and the lowest in China (4.16%) [3]. A meta‐analysis reported that the prevalence of GERD in Iran is 43.07%, which is markedly higher than the worldwide percentage [4]. Symptom‐based epidemiological data indicate that many people may be living with GERD symptoms globally, and this burden has been steadily rising over recent decades due to demographic changes, lifestyle factors, and increasing obesity rates [4, 5].

Risk factors contributing to GERD include advanced age, high body mass index (BMI), smoking, anxiety, low physical activity, and diet‐related factors such as the acidity of food, meal portion sizes, eating close to bedtime, and physical activity after meals [6, 7]. Vitamin B6 is a water‐soluble micronutrient that functions as a coenzyme in multiple metabolic pathways, including one‐carbon metabolism, which is essential for amino acid metabolism, nucleotide synthesis, and cellular methylation processes [8]. Through its role as pyridoxal‐5′‐phosphate, vitamin B6 contributes to pathways involved in tissue integrity and cellular homeostasis, processes that may be relevant to gastrointestinal mucosal function and disease susceptibility [9].

Recent studies suggest that vitamin B6 may influence GERD risk. For instance, higher intake of vitamin B6 was reported to be inversely associated with the risk of reflux esophagitis (RE) as well as esophageal adenocarcinoma (EAC) and Barrett's esophagus (BE) [10]. Another study showed that a high intake of vitamin B6 was associated with a reduced risk of non‐erosive reflux disease (NERD) [11]. Although vitamin B6 has been suggested to exert beneficial effects in some contexts, its role as a coenzyme in pathways related to histamine synthesis and neurotransmitter metabolism raises the possibility that higher vitamin B6 intake may exacerbate gastroesophageal reflux symptoms in certain individuals, particularly depending on dietary context and reflux phenotype [12, 13]. So, this study aimed to evaluate the association between dietary vitamin B6 intake and the risk of GERD, providing new insight into the role of micronutrients in GERD pathophysiology.

## 2 Methods

### 2.1 Study Design and Participants

This case–control study was conducted in Tehran, Iran, in 2024 and included 450 adult women, comprising 150 participants with GERD and 300 control subjects. Participants were selected through simple random sampling from adults aged 35–70 years in Tehran, Iran. The inclusion criteria were women aged 35–70 years, absence of a family history of GERD, no current use of medications known to influence GERD symptoms, and no consumption of vitamin B6 supplements. Exclusion criteria included the inability to provide necessary data and declined continued participation.

GERD was defined based on self‐reported symptoms of heartburn and/or acid reflux occurring more than once per week. Symptom frequency data were collected during in‐person interviews with trained researchers. Demographic, social, and medical data were collected during face‐to‐face interviews. Physical activity levels were assessed using a validated short form of the International Physical Activity Questionnaire (IPAQ) [14]. Participants' body weight was measured to the nearest 0.1 kg using a calibrated scale (Seca 755) with light clothing and without shoes. Height was measured using a portable stadiometer accurate to 0.1 cm (Seca 204). Body mass index (BMI) was calculated as weight in kilograms divided by height in meters squared.

### 2.2 Dietary Intake

Dietary intake data were collected using a semi‐quantitative food frequency questionnaire (FFQ), previously validated for reliability and accuracy in the Iranian adult population [15]. The FFQ was designed to assess habitual dietary intake over the previous year and included a comprehensive list of commonly consumed Iranian foods with predefined portion sizes. Participants reported the frequency of consumption of each food item on a daily, weekly, or monthly basis. Reported intakes were converted into grams per day, and daily vitamin B6 intake was estimated using Nutritionist IV software (First Databank, San Bruno, CA, USA), modified to include Iranian food composition data.

### 2.3 Statistical Analysis

Quantitative data were analyzed using independent *t*‐tests or their non‐parametric equivalent (Mann–Whitney U test), while chi‐square tests were applied for qualitative variables. Logistic regression models were used to assess the association between GERD and dietary intake of vitamin B6 employing the following models: Model 1 was adjusted for age; Model 2 was additionally adjusted for education, marital status, occupation, physical activity, and BMI; and Model 3 was further adjusted for energy intake. All analyses were performed using SPSS software version 21, with a significance level of *p* < 0.05.

## 3 Results

The general characteristics of the participants including age, height, weight, BMI, history of diabetes and blood pressure, marital status, educational qualification, and complete blood count (CBC) analysis are presented in Table 1. The case group had a higher weight (70.05 ± 11.36 vs. 73.32 ± 11.57, *p* = 0.010), BMI (28.67 ± 4.23 vs. 30.04 ± 4.22, *p* = 0.003), diastolic blood pressure (69.30 ± 9.13 vs. 72.19 ± 8.46, *p* = 0.003), and systolic blood pressure (107.67 ± 13.77 vs. 111.01 ± 14.49, *p* = 0.033).

**TABLE 1:** Characteristics of the participants.

		Controls	Cases	*p*
Age (years)		49.26 ± 8.53	49.85 ± 8.05	0.513
Height (cm)		156.20 ± 5.29	156.12 ± 5.87	0.885
Weight (kg)		70.05 ± 11.36	73.32 ± 11.57	0.010
BMI (kg/m^2^)		28.67 ± 4.23	30.04 ± 4.22	0.003
Physical activity (hours)		1.58 ± 1.58	1.36 ± 1.34	0.174
Right DBP (mm Hg)		69.30 ± 9.13	72.19 ± 8.46	0.003
Right SBP (mm Hg)		107.67 ± 13.77	111.01 ± 14.49	0.033
Diabetes (*n* (%))		30 (13.8%)	23 (17.0%)	0.404
Hypertension (*n* (%))		42 (19.3%)	39 (28.9%)	0.050
Marital state (*n* (%))	Separated	1 (0.5%)	0 (0.0%)	0.409
	Married	196 (89.9%)	121 (89.6%)	
	Single	18 (8.3%)	9 (6.7%)	
	Widowed	3 (1.4%)	5 (3.7%)	
Last education (*n* (%))	Diploma	56 (25.7%)	42 (31.1%)	0.172
	Bachelor's	34 (15.6%)	26 (19.3%)	
	Master's	54 (24.8%)	25 (18.5%)	
	PhD	7 (3.2%)	1 (0.7%)	
	< Diploma	10 (4.6%)	4 (3.0%)	
	> PhD	0 (0.0%)	2 (1.5%)	
	Unknown	57 (26.1%)	35 (25.9%)	

Abbreviations: BMI = body mass index; Right DBP = right hand diastolic blood pressure; Right SBP = right hand systolic blood pressure.

The dietary intake of the participants is presented in Table 2. Cases showed a slightly higher intake of vitamin B6 than controls (1.95 ± 0.52 vs. 1.84 ± 0.61 mg/day), although the difference did not reach statistical significance (*p* = 0.078). Intakes of energy, macronutrients, cholesterol, fatty acid subtypes, and other micronutrients did not differ significantly between cases and controls (all *p* > 0.05). However, when vitamin B6 intake was categorized into tertiles, a significant association with GERD was observed.

**TABLE 2:** Daily nutrient intake of the study participants.

	Controls	Cases	*p*
Calories (kcal)	2586.54 ± 539.76	2592.64 ± 315.84	0.894
Protein (g)	83.81 ± 26.94	84.50 ± 18.90	0.778
Carbohydrates (g)	369.84 ± 75.15	372.84 ± 45.21	0.640
Total Fat (g)	93.52 ± 24.22	93.44 ± 17.30	0.973
Cholesterol (mg)	272.48 ± 142.56	262.56 ± 134.21	0.520
Saturated fat (g)	28.89 ± 13.57	30.62 ± 10.10	0.172
Monounsaturated fat (g)	33.31 ± 12.93	34.05 ± 13.15	0.606
Polyunsaturated fat (g)	19.60 ± 7.84	18.57 ± 7.57	0.223
Omega‐3 fatty acids (g)	1.25 ± 0.71	1.31 ± 0.69	0.449
Omega‐6 fatty acids (g)	6.33 ± 5.58	6.09 ± 5.11	0.727
Vitamin B6 (mg)	1.84 ± 0.61	1.95 ± 0.52	0.078

The association between GERD and dietary intake of vitamin B6 is presented in Table 3. In Model 1, adjusting for age and vitamin B6 intake, those in the highest tertile of vitamin B6 intake had approximately twice the odds of GERD compared with those in the lowest tertile (OR = 1.98, 95% CI: 1.12–3.51, *p* = 0.019). People in the second tertile, however, had no statistically significant odds compared to the lowest tertile. After adjusting for education, marital status, occupation, physical activity, and BMI (Model 2), the association between the highest tertile of vitamin B6 intake and GERD remained significant (OR = 2.05, 95% CI: 1.10–3.83, *p* = 0.025). In the fully adjusted model (Model 3), with consideration of total calorie intake, the results did not change (OR = 2.02, 95% CI: 1.06–3.81, *p* = 0.031).

**TABLE 3:** The association of GERD and dietary intake of vitamin B6.

	Tertile 1	Tertile 2			Tertile 3		
		OR	95% CI	*p*	OR	95% CI	*p*
Model 1	Reference	1.264	0.721–2.216	0.412	1.983	1.121–3.508	0.019
Model 2	Reference	1.291	0.712–2.341	0.400	2.048	1.095–3.831	0.025
Model 3	Reference	1.303	0.710–2.393	0.393	2.015	1.064–3.814	0.031

*Note:* Model 1: adjusted for age; Model 2: additionally adjusted for education, marital status, occupation, physical activity, and BMI; Model 3: further adjustment for calorie intake.

## 4 Discussion

This study indicated a higher risk of gastroesophageal reflux disease (GERD) among Iranian adults with higher intakes of vitamin B6. In the present study, although the mean vitamin B6 intake did not differ significantly between cases and controls, a significant association between GERD and vitamin B6 was observed when vitamin B6 intake was analyzed across tertiles.

This result on the association between GERD and dietary intake of vitamin B6 differs from some previous studies that have reported inverse or null associations between vitamin B6 intake and reflux‐related outcomes. For example, in contrast to our result, one study found that higher intake of vitamin B6, along with other antioxidants like vitamins A, B2, and folic acid, was linked to a 22%–32% decrease in the risk of non‐erosive reflux disease (NERD) [11]. In our study, however, diagnosis was based on symptom reporting rather than clinical evaluation, which could partly explain the differences in findings. Additionally, a randomized controlled trial reported that a supplement containing vitamin B6, among other nutrients, was associated with complete symptom regression after 40 days, whereas symptom regression was observed in 65.7% of participants receiving omeprazole [16]. Through its role in one‐carbon metabolism, vitamin B6 was supposed to contribute to DNA synthesis and repair, which may be important for preserving esophageal mucosal integrity [17]. While previous research has highlighted the potential role of different types of micronutrients in gastrointestinal health, our study contributes by focusing specifically on vitamin B6.

Moreover, some studies have not established any relationship between GERD and vitamin B6 [11, 18, 19]. The potential effect of vitamin B6 may depend on its dietary sources and the broader dietary context, which can vary considerably across populations. For instance, higher vitamin B6 intake in this population may serve as a marker of broader dietary patterns, rather than an independent effect, particularly patterns involving higher consumption of animal protein and fat, which have been linked to GERD symptoms [20]. In contrast, studies reporting inverse associations often describe vitamin B6 intake predominantly derived from plant‐based foods, including fruits, vegetables, and whole grains, which have been independently associated with a lower risk or severity of reflux‐related symptoms [21]. Dietary vitamin B6 is obtained from a wide range of foods, including meat, poultry, fish, legumes, nuts, whole grains, and starchy vegetables such as potatoes; however, the relative contribution of these sources to total vitamin B6 intake may vary across populations depending on country‐specific dietary patterns and food consumption habits [22]. Furthermore, these discrepancies may be explained by differences in dietary assessment methods and definitions of reflux‐related outcomes.

The mechanisms through which vitamin B6 intake may adversely influence GERD risk remain unclear. Vitamin B6, primarily in its active form pyridoxal‐5′‐phosphate (PLP), acts as a coenzyme for numerous PLP‐dependent enzymes involved in amino acid metabolism and the synthesis of bioactive amines and neurotransmitters [23]. From a biological perspective, two non‐exclusive mechanisms may be relevant to GERD. First, PLP is required for histidine decarboxylase, the enzyme responsible for histamine synthesis [12]. Histamine plays a central role in the regulation of gastric acid secretion, particularly via H2 receptor signaling in the stomach [24], and increased availability of PLP could theoretically facilitate histamine production in settings where this pathway is upregulated. Second, PLP serves as a coenzyme for enzymes involved in serotonin and other neurotransmitter pathways, which have been implicated in gastrointestinal motility, visceral sensitivity, and transient lower esophageal sphincter (LES) relaxations [13]. Nevertheless, direct evidence linking dietary vitamin B6 intake to these mechanisms in the pathogenesis of GERD is limited, and these considerations should be regarded as hypothesis‐generating rather than indicative of a causal relationship [13, 25].

Our study has several strengths, including its focus on an understudied nutrient and the use of a validated and comprehensive dietary assessment tool and multivariable adjustment for key confounders. From a clinical perspective, these findings may help discourage the unnecessary use of vitamin B6 supplements in individuals with GERD, particularly in the absence of clear deficiency or evidence of benefit, and reinforce the importance of focusing on overall dietary patterns rather than routine micronutrient supplementation. However, there are limitations. The use of a FFQ may introduce recall bias and measurement error and the population was limited to Iranian adults aged 35–70 years, potentially limiting generalizability. The case–control design limits causal inference and does not allow the temporal relationship between dietary vitamin B6 intake and GERD to be established. Although a symptom‐based definition was used, the absence of endoscopic or physician‐confirmed diagnosis may have resulted in non‐differential misclassification. In addition, regional and cultural dietary habits may have influenced the sources of vitamin B6 intake, and this study did not differentiate between specific dietary sources of vitamin B6. Furthermore, the interplay between various nutrients and lifestyle factors makes it difficult to isolate the independent effect of vitamin B6.

In conclusion, our findings suggest that dietary vitamin B6 intake may be linked to GERD risk, potentially through mechanisms involving neurotransmitter synthesis, LES function, and mucosal integrity. Further longitudinal and interventional studies are needed to clarify these associations, consider confounding lifestyle factors, and explore the physiological mechanisms underlying the relationship between micronutrients and GERD.

## 5 Conclusion

In this case–control study, higher dietary vitamin B6 intake was associated with increased odds of GERD, and this association persisted after adjustment for multiple confounders. These results suggest a potential link between vitamin B6 intake and GERD; however, the case–control design precludes any causal inference. Moreover, since GERD was defined based on self‐reported symptoms, these findings should be considered hypothesis‐generating and warrant confirmation in prospective studies. The mechanisms underlying the relationship between dietary micronutrients and GERD remain unclear, highlighting the need for further prospective and mechanistic research that considers dietary sources and population‐specific factors.

## Funding

Shahid Beheshti University of Medical Sciences, Tehran, Iran, provided financial support for the investigation (Code 43014454).

## Ethics Statement

All methods were conducted in compliance with the relevant guidelines and regulations set by the Ethical Committee of Shahid Beheshti University of Medical Sciences, Tehran, Iran (Code: IR.SBMU.RETECH.REC.1404.112). Informed consent was obtained from all participants, and they signed consent forms before taking part in the study.

## Conflicts of Interest

The authors declare no conflicts of interest.

## Data Availability

The datasets used and/or examined in the present investigation may be obtained from the corresponding author upon reasonable request.

## References

[1] P. O. Katz , L. B. Gerson , and M. F. Vela , “Guidelines for the Diagnosis and Management of Gastroesophageal Reflux Disease,” American Journal of Gastroenterology 108, no. 3 (2013): 308–328. 23419381 10.1038/ajg.2012.444

[2] Y. Yu , S. Wen , S. Wang , et al., “Reflux Characteristics in Patients With Gastroesophageal Reflux‐Related Chronic Cough Complicated by Laryngopharyngeal Reflux,” Annals of Translational Medicine 7, no. 20 (2019): 529. 31807511 10.21037/atm.2019.09.162 PMC6861741

[3] J. S. Nirwan , S. S. Hasan , Z.‐U.‐D. Babar , B. R. Conway , and G. MUJSr , “Global Prevalence and Risk Factors of Gastro‐Oesophageal Reflux Disease (GORD): Systematic Review With Meta‐Analysis,” Scientific Reports 10, no. 1 (2020): 5814. 32242117 10.1038/s41598-020-62795-1 PMC7118109

[4] M. Karimian , H. Nourmohammadi , M. Salamati , et al., “Epidemiology of Gastroesophageal Reflux Disease in Iran: A Systematic Review and Meta‐Analysis,” BMC Gastroenterology 20, no. 1 (2020): 297. 32928126 10.1186/s12876-020-01417-6 PMC7488684

[5] K. Tuerxun , M. Balati , M. Aimaiti , et al., “Epidemiological Investigation, Extraesophageal Symptoms and Risk Factors of Gastroesophageal Reflux Disease in Kashgar, Xinjiang, China,” 13, no. 12 (2021): 14186. PMC8748132 35035764

[6] L. H. Eusebi , A. Telese , G. G. Cirota , et al., “Effect of Gastro‐Esophageal Reflux Symptoms on the Risk of Barrett's Esophagus: A Systematic Review and Meta‐Analysis,” Journal of Gastroenterology and Hepatology 37, no. 8 (2022): 1507–1516. 35614860 10.1111/jgh.15902 PMC9543729

[7] Z. Zheng , H. Nordenstedt , N. L. Pedersen , J. Lagergren , and W. Ye , “Lifestyle Factors and Risk for Symptomatic Gastroesophageal Reflux in Monozygotic Twins,” Gastroenterology 132, no. 1 (2007): 87–95. 17241862 10.1053/j.gastro.2006.11.019 PMC2230637

[8] P. M. Ueland , A. Ulvik , L. Rios‐Avila , Ø. Midttun , and J. F. Gregory , “Direct and Functional Biomarkers of Vitamin B6 Status,” Annual Review of Nutrition 35, no. 1 (2015): 33–70. 10.1146/annurev-nutr-071714-034330 PMC5988249 25974692

[9] V. Lotto , S.‐W. Choi , and S. Friso , “Vitamin B6: A Challenging Link Between Nutrition and Inflammation in CVD,” British Journal of Nutrition 106, no. 2 (2011): 183–195. 21486513 10.1017/S0007114511000407

[10] L. Sharp , A. E. Carsin , M. M. Cantwell , et al., “Intakes of Dietary Folate and Other B Vitamins Are Associated With Risks of Esophageal Adenocarcinoma, Barrett's Esophagus, and Reflux Esophagitis,” 143, no. 12 (2013): 1966–1973. 10.3945/jn.113.174664 24132576

[11] S. Y. Nam , B. J. Park , Y.‐A. Cho , and K. H. Ryu , “Gender‐Specific Effect of Micronutrient on Non‐Erosive Reflux Disease and Erosive Esophagitis,” Journal of Neurogastroenterology and Motility 25, no. 1 (2019): 82. 30646479 10.5056/jnm18114 PMC6326192

[12] N. Hirasawa , “Expression of Histidine Decarboxylase and Its Roles in Inflammation,” International Journal of Molecular Sciences 20, no. 2 (2019): 376. 30654600 10.3390/ijms20020376 PMC6359378

[13] X. Yang , J. Lou , W. Shan , et al., “Pathophysiologic Role of Neurotransmitters in Digestive Diseases,” Frontiers in Physiology 12 (2021): 567650. 34194334 10.3389/fphys.2021.567650 PMC8236819

[14] M. B. Moghaddam , F. B. Aghdam , M. A. Jafarabadi , et al., “The Iranian Version of International Physical Activity Questionnaire (IPAQ) in Iran: Content and Construct Validity, Factor Structure, Internal Consistency and Stability,” World Applied Sciences Journal 18, no. 8 (2012): 1073–1080.

[15] P. Mirmiran , F. H. Esfahani , Y. Mehrabi , et al., “Reliability and Relative Validity of an FFQ for Nutrients in the Tehran Lipid and Glucose Study,” Public Health Nutrition 13, no. 5 (2010): 654–662. 19807937 10.1017/S1368980009991698

[16] R. de Souza Pereira , “Regression of Gastroesophageal Reflux Disease Symptoms Using Dietary Supplementation With Melatonin, Vitamins and Aminoacids: Comparison With Omeprazole,” Journal of Pineal Research 41, no. 3 (2006): 195–200. 16948779 10.1111/j.1600-079X.2006.00359.x

[17] J. de Wit , “The Role of Vitamin B6 in One‐Carbon Metabolism, Oxidative Stress, Immune System Regulation and Carcinogenesis,” (2011).

[18] N. Lakananurak , P. Pitisuttithum , P. Susantitaphong , et al., “The Efficacy of Dietary Interventions in Patients With Gastroesophageal Reflux Disease: A Systematic Review and Meta‐Analysis of Intervention Studies,” Nutrients 16, no. 3 (2024): 464. 38337748 10.3390/nu16030464 PMC10857327

[19] K. Komolafe , T. R. Komolafe , O. O. Crown , et al., “Natural Products in the Management of Gastroesophageal Reflux Disease: Mechanisms, Efficacy, and Future Directions,” Nutrients 17, no. 6 (2025): 1069. 40292509 10.3390/nu17061069 PMC11944625

[20] L. Baroni , C. Bonetto , I. Solinas , et al., “Diets Including Animal Food Are Associated With Gastroesophageal Reflux Disease,” European Journal of Investigation in Health, Psychology and Education 13, no. 12 (2023): 2736–2746. 38131888 10.3390/ejihpe13120189 PMC10742960

[21] J. I. Bucan , T. Braut , A. Krsek , V. Sotosek , and L. Baticic , “Updates in Gastroesophageal Reflux Disease Management: From Proton Pump Inhibitors to Dietary and Lifestyle Modifications,” Gastrointestinal Disorders 7, no. 2 (2025): 33.

[22] K. M. Fairfield and R. H. Fletcher , “Vitamins for Chronic Disease Prevention in Adults: Scientific Review,” JAMA 287, no. 23 (2002): 3116–3126. 12069675 10.1001/jama.287.23.3116

[23] M. H. Al Mughram , M. S. Ghatge , G. E. Kellogg , and M. K. Safo , “Elucidating the Interaction Between Pyridoxine 5′‐Phosphate Oxidase and Dopa Decarboxylase: Activation of B6‐Dependent Enzyme,” International Journal of Molecular Sciences 24, no. 1 (2022): 642. 36614085 10.3390/ijms24010642 PMC9820991

[24] H. L. Waldum , Ø. F. Sørdal , and P. G. Mjønes , “The Enterochromaffin‐Like [ECL] Cell—Central in Gastric Physiology and Pathology,” International Journal of Molecular Sciences 20, no. 10 (2019): 2444. 31108898 10.3390/ijms20102444 PMC6567877

[25] S. Heidarzadeh‐Asl , M. Maurer , A. Kiani , D. Atiakshin , P. S. Skov , and D. Elieh‐Ali‐Komi , “Novel Insights on the Biology and Immunologic Effects of Histamine: A Road Map for Allergists and Mast Cell Biologists,” Journal of Allergy and Clinical Immunology 155, no. 4 (2025): 1095–1114. 39734034 10.1016/j.jaci.2024.12.1081

